# High-Intensity Interval Training: A Potential Exercise Countermeasure During Human Spaceflight

**DOI:** 10.3389/fphys.2019.00581

**Published:** 2019-05-22

**Authors:** Christopher Hurst, Jonathan P. R. Scott, Kathryn L. Weston, Matthew Weston

**Affiliations:** ^1^ Institute of Neuroscience, Newcastle University, Newcastle upon Tyne, United Kingdom; ^2^ NIHR Newcastle Biomedical Research Centre, Newcastle upon Tyne Hospitals NHS Foundation Trust and Newcastle University, Newcastle upon Tyne, United Kingdom; ^3^ KBRwyle GmbH, Cologne, Germany; ^4^ Space Medicine Office, European Astronaut Centre, European Space Agency (ESA), Cologne, Germany; ^5^ School of Health and Social Care, Teesside University, Middlesbrough, United Kingdom

**Keywords:** high-intensity interval training, cardiorespiratory fitness, neuromuscular fitness, human spaceflight, microgravity, physical performance, exercise countermeasure

## Abstract

High-intensity interval training (HIT) is an effective approach for improving a range of physiological markers associated with physical fitness. A considerable body of work has demonstrated substantial improvements in cardiorespiratory fitness following short-term training programmes, while emerging evidence suggests that HIT can positively impact aspects of neuromuscular fitness. Given the detrimental consequences of prolonged exposure to microgravity on both of these physiological systems, and the potential for HIT to impact multiple components of fitness simultaneously, HIT is an appealing exercise countermeasure during human spaceflight. As such, the primary aim of this mini review is to synthesize current terrestrial knowledge relating to the effectiveness of HIT for inducing improvements in cardiorespiratory and neuromuscular fitness. As exercise-induced fitness changes are typically influenced by the specific exercise protocol employed, we will consider the effect of manipulating programming variables, including exercise volume and intensity, when prescribing HIT. In addition, as the maintenance of HIT-induced fitness gains and the choice of exercise mode are important considerations for effective training prescription, these issues are also discussed. We conclude by evaluating the potential integration of HIT into future human spaceflight operations as a strategy to counteract the effects of microgravity.

## Introduction

The prolonged exposure to microgravity (μG) and the space environment associated with human spaceflight necessitates effective countermeasures to manage the multi-system adaptation that occurs. These adaptations are both short term, including headache, drowsiness, nausea, vomiting, and dizziness, collectively referred to as “space motion sickness” (Ortega and Harm, 2008), and longer term, including fluid redistribution and reductions in maximal oxygen uptake (VO_2max_), muscle size and strength, and bone mineral density (BMD) (Demontis et al., 2017).

Exercise training is a fundamental strategy for managing adaptation to spaceflight; however, the potential physical (size and internal dimensions of vehicle/habitats), logistical (supply of food and water and device maintenance/repair), and operational (time for exercise, interference with other crewmembers’ work) constraints of future space exploration missions highlight a need for alternate approaches to counteracting μG-induced changes (Scott et al., 2019). High-intensity interval training (HIT), involving repeated bouts of intense exercise, interspersed with periods of rest or lower intensity active recovery, is a widely used training approach with demonstrated efficacy for inducing physiological adaptation across a range of outcomes. As an exercise countermeasure, HIT may offer several operational advantages, including; (1) substantial physiological stimulus possible in a short time period; (2) the potential to impact multiple components of fitness simultaneously; (3) is typically performed using a single exercise mode; (4) an ability to target upper- and lower-body function. This mini-review aims to highlight the potential for HIT as an exercise countermeasure during human spaceflight by summarizing the terrestrial evidence base relating to its effectiveness and considering exercise programming variables in the context of spaceflight.

## High-Intensity Interval Training

Despite intensifying scientific enquiry over the last 15–20 years, HIT is not a new approach to exercise training (Billat, 2001). Although terminology varies, HIT can be: high-intensity interval training (HIT), performed at “near maximal” or “submaximal” intensity (≥80% maximal heart rate), or sprint interval training (SIT), often described as low-volume HIT, characterized by efforts performed at “all out” or “supramaximal” intensity (≥100% VO_2max_) (Weston et al., 2014a; MacInnis and Gibala, 2017). Despite broad protocol dichotomization, HIT exists on a continuum, encompassing a wide spectrum of exercise intensities, with longer duration HIT intervals (e.g., Wisloff et al., 2007) at the lower end and SIT (e.g., Gibala et al., 2006) at the upper end. As exercise intensity is a key mediator of training adaptation (Shephard, 1968), it may be the intense stimulus induced by HIT is a potent catalyst for physiological remodelling (MacInnis and Gibala, 2017). Despite a predominant focus on VO_2max_ improvement, the intensity of HIT places considerable demands on both the aerobic and anaerobic energy systems and the neuromuscular system (Buchheit and Laursen, 2013a,b), suggesting potential for adaptation across multiple physiological systems.

## Effectiveness of High-Intensity Interval Training

### Cardiorespiratory Fitness

Numerous interventions (e.g., Helgerud et al., 2007; Burgomaster et al., 2008; Matsuo et al., 2014; Astorino et al., 2017) demonstrated HIT as a potent strategy for improving VO_2max_. These experimental findings have been corroborated in several meta-analyses in healthy (Bacon et al., 2013; Sloth et al., 2013; Weston et al., 2014b; Milanovic et al., 2015) and clinical populations (Weston et al., 2014a; Liou et al., 2016). Compared with moderate intensity continuous training (MICT), HIT may elicit adaptations of a similar (Gibala et al., 2006; Burgomaster et al., 2008; Scribbans et al., 2014) or even greater magnitude (Helgerud et al., 2007; Daussin et al., 2008; Matsuo et al., 2014), despite a substantially reduced time commitment. Previous work has reported large improvements in VO_2max_ following HIT (Mean ± SD; 22.5 ± 12.2%) and SIT (16.7 ± 11.6%), compared with a moderate improvement (10.0 ± 8.9%) following continuous training (Matsuo et al., 2014), while a recent meta-analysis demonstrated a possibly small beneficial effect for HIT on VO_2max_ (1.2 ml kg^−1^ min^−1^; 95% confidence limits ±0.9 ml kg^−1^ min^−1^) when compared with continuous endurance training (Milanovic et al., 2015). It may be that the underlying physiological mechanisms differ between HIT and MICT (Daussin et al., 2008), although this remains to be fully determined.

Exercise at both ends of the intensity continuum, and that representing the middle ground (e.g., Little et al., 2010), can induce substantial (e.g., 10–15%) improvements in VO_2max_ following short-term training programmes (MacPherson et al., 2011; Metcalfe et al., 2012; Matsuo et al., 2014). Nevertheless, participant-related factors (e.g., baseline fitness; Weston et al., 2014b) and protocol-related factors (e.g., repetition duration; Bacon et al., 2013) moderate responses, suggesting that effective manipulation of programming variables is necessary to maximize physiological adaptation (Buchheit and Laursen, 2013a). While mechanisms responsible for HIT-induced improvements in cardiorespiratory fitness remain elusive, both peripheral (e.g., increased mitochondrial content and function) and central adaptations (e.g., increased cardiac output) may contribute to increased VO_2max_ (Daussin et al., 2007; Burgomaster et al., 2008; Jacobs et al., 2013; Astorino et al., 2017).

### Neuromuscular Fitness

The intensity of HIT induces a substantial acute neuromuscular load (Buchheit and Laursen, 2013a) and high levels of muscle fiber recruitment (Sale, 1987), therefore providing a stimulus for neuromuscular adaptation (Creer et al., 2004; Martinez-Valdes et al., 2017). Although resistance training represents the primary strategy for improving muscle morphology, previous investigations demonstrated HIT-induced increases in lean- or fat-free mass (Gillen et al., 2013; Robinson et al., 2017; Sculthorpe et al., 2017) and muscle cross-sectional area (Osawa et al., 2014). Increases in protein synthesis (Bell et al., 2015) and satellite cell activity (Nederveen et al., 2015) may contribute to these observed changes. These findings are not universal however (Nybo et al., 2010), and the potential for HIT to increase muscle mass remains largely unknown.

Substantial improvements in mean and peak power output (PPO) of ~5–20% have been observed following SIT (Burgomaster et al., 2005, 2006; Astorino et al., 2011; Zelt et al., 2014; Sculthorpe et al., 2017), potentially mediated by changes in anaerobic enzyme activity (MacDougall et al., 1998; Rodas et al., 2000). However, power output determined during short-duration cycling bouts (e.g., Wingate test) may primarily represent metabolic not neuromuscular power. Nonetheless, emerging evidence suggests that HIT increases explosive muscular power, assessed *via* leg extension (Hurst et al., 2018) and standing broad jump (Buckley et al., 2015). Improvements in muscle strength following HIT also occur (McRae et al., 2012; Buckley et al., 2015; Martinez-Valdes et al., 2017) with small-moderate increases (~7%) in knee extensor strength following six sessions of cycle-based HIT performed at 100% PPO (Martinez-Valdes et al., 2017). These findings reaffirm the potential for HIT as a training strategy capable of improving cardiorespiratory and neuromuscular fitness simultaneously.

## Programming Considerations for High-Intensity Interval Training During Spaceflight

While HIT can simultaneously improve cardiorespiratory and neuromuscular fitness, acute training responses and subsequent adaptations are determined by the interaction of several programming variables (Buchheit and Laursen, 2013a,b; MacInnis and Gibala, 2017). The following section discusses programming considerations relevant to the operational use and potential advantages of HIT during spaceflight.

### Exercise Volume

Low-volume HIT, typically involving four to six repetitions of 30–60 s exercise performed at “all-out” intensity, induces substantial improvements in cardiorespiratory fitness (Sloth et al., 2013; Weston et al., 2014b) and may offer potential for rapid fitness gains in a short time period. However, despite the potent effects of this training stimulus, the intensive nature of this exercise protocol necessitates substantial recovery periods between intervals (~4 min), meaning that session duration is often ~30 minutes. Reducing the volume of exercise does not necessarily lessen the magnitude of adaptation following SIT, and improvements in VO_2max_ can be enhanced with fewer repetitions (Vollaard et al., 2017). For example, a protocol of 3 × 20 s all out cycle sprints performed three times per week for 6 weeks (Gillen et al., 2014) or 12 weeks (Gillen et al., 2016) increased peak oxygen uptake (VO_2peak_) by 12 and 19%, respectively. Reducing exercise volume further, improvements of 10–15% in VO_2peak_ can occur following 6 weeks of three sessions per week involving only 2 × 20 s all out sprints (Metcalfe et al., 2012, 2016). Importantly, a reduced exercise volume does not appear to have a detrimental effect on anaerobic, as well as aerobic performance, given that improvements in PPO were not different following 2–4 weeks of SIT intervals of either 15 or 30 s (Zelt et al., 2014) or 10 or 30 s (Hazell et al., 2010) duration. Even with a reduced exercise volume, HIT maintains the potential to induce rapid fitness gains.

Exercise training programmes typically involve a combination of resistance and endurance training and are termed “concurrent” (Fyfe et al., 2014) or “combined” training (Hurst et al., 2019). Although resistance and endurance training represent effective strategies for improving muscular and cardiorespiratory fitness respectively, concurrent training may induce an “interference effect” whereby improvements in muscular fitness are attenuated compared with performing resistance training alone (Fyfe et al., 2014). Incorporating SIT into a concurrent training programme may help to mitigate any observed interference effects (Cantrell et al., 2014), as these may largely be exercise volume rather than intensity dependent (Fyfe et al., 2016).

### Differentiation of High-Intensity Interval Training

As HIT incorporates a broad spectrum of intensities, performing exercise across this range is an effective strategy to induce a differential adaptive response (Barnes et al., 2013; Rønnestad et al., 2015). Exercise bout duration represents a key programming variable because of the inverse relationship between duration and intensity (i.e., shorter intervals typically involve higher intensity exercise). Therefore, manipulating exercise duration alters energy system contribution (Gastin, 2001) as well as the degree of neuromuscular loading (Buchheit and Laursen, 2013b). Shorter (30 s) compared with long duration cycle-based intervals (300 s) have been demonstrated to result in a higher training intensity (363 ± 32 W vs. 324 ± 42 W) and lead to significant increases in VO_2max_ (8.7 ± 5.0%) and PPO (8.5 ± 5.2%) (Rønnestad et al., 2015). Furthermore, following uphill running-based HIT, improvements in aerobic fitness and performance variables were optimal around the middle intensity (100% velocity at VO_2max_; 10% gradient; 1:2 work:rest ratio) with increases in neuromuscular measures (e.g., peak power, maximum rate of force development) greatest at the highest intensity (Barnes et al., 2013). Repeated-sprint training (RST), typically defined as a series of short sprints (3–7 s in duration), separated by recovery periods of less than 60 s (Buchheit and Laursen, 2013a), is another HIT derivative at the highest end of the intensity spectrum. As with SIT, RST induces considerable acute metabolic and neuromuscular demands (Buchheit and Laursen, 2013b), thereby highlighting potential as a multicomponent training tool. This supposition was supported in a recent meta-analysis that reported clear beneficial effects of RST on measures of countermovement jump height, sprint times, repeated sprint ability, and high-intensity running performance (Taylor et al., 2015). Manipulating HIT exercise intensity therefore promotes a differential training response, with these findings further demonstrating potential for HIT as a combined training tool for inducing adaptation across multiple physiological systems. Ultimately, varied HIT prescription within a training programme (e.g., Wright et al., 2016) is necessary to maximize metabolic and neuromuscular adaptations (Buchheit and Laursen, 2013a,b).

### Maintenance of High-Intensity Interval Training-Induced gains

Although short-term fitness gains are well documented following HIT, maintaining fitness over an extended time period represents another challenge. To date however, only a limited number of studies evaluated the effects of manipulating session frequency on the maintenance of HIT-induced fitness improvements. Following a 2-week SIT intervention, which increased VO_2max_ (3%) and high-intensity intermittent running performance (17%), participants completed a single weekly SIT session for 5 weeks (Macpherson and Weston, 2015). Interestingly, this maintenance phase induced a 4.2% improvement in VO_2max_, indicating that reduced training frequency can be an effective strategy to maintain SIT-induced fitness improvements (Macpherson and Weston, 2015). In another investigation, performing 24 HIT sessions at either moderate frequency (MF; three sessions per week) or high frequency (HF; eight sessions per week) led to a 10.7% increase in VO_2max_ in the MF group with no statistically significant improvement (3.0%) in the HF group (Hatle et al., 2014). Following the intervention, participants completed a 9-week detraining period involving no training with both groups demonstrating increased VO_2max_ at 12 days post-intervention and a return to baseline 4 weeks after highest measurement (Hatle et al., 2014). These data support the idea that lower frequency training may be as effective as higher frequency training for maintaining fitness, although there remains only limited evidence to support this assertion, particularly in well-trained individuals.

### Exercise Mode

Traditionally, HIT has been delivered using a single exercise mode with treadmill walking/running and cycle ergometry, the most commonly used approaches. However, despite the logistical advantages of this approach, these exercise modes deliver a predominantly lower-body training stimulus. In the context of spaceflight, this is likely to be suboptimal because the performance profile of astronauts necessitates a synergy of upper- and lower-body fitness. Recently, however, there has been an increased desire to move beyond the exercise modes typically associated with HIT. Alternative exercise modes for performing HIT include body-weight resistance exercise (McRae et al., 2012), non-weight bearing all-extremity ergometers (Hwang et al., 2016), hydraulic resistance machines (Hurst et al., 2018), a combination of strength and endurance exercises (Buckley et al., 2015), and high-intensity circuit-type training (Sperlich et al., 2017). These modes provide a whole-body training stimulus, inducing substantial improvements in VO_2peak_ (~8%), lower-body muscle power (6–15%), upper- and lower-body 1RM strength (27%), and muscular endurance (40–280%) (McRae et al., 2012; Buckley et al., 2015), following short-term training programmes.

As well as the need for upper- and lower-body fitness, exercise interventions delivered using a single exercise mode are desirable because of physical constraints during spaceflight. Performing combined upper- and lower-body HIT using a hydraulic resistance machine for 12 weeks improves explosive leg power (~10%) and predicted VO_2max_ (8.4%) (Hurst et al., 2018), while 8 weeks of HIT performed using a non-weight-bearing ergometer improves aerobic fitness (11%) and cardiac systolic function (Hwang et al., 2016). While these findings are encouraging, it should be noted that both of these studies involved older adults with relatively low baseline fitness who typically demonstrate greater training-induced improvements. Collectively, however, these data highlight potential for innovative approaches to training delivery and should encourage researchers to explore alternative exercise modes.

## Integration into Current and/or Future Human Spaceflight Operations

Interval exercise during spaceflight is not new, having been previously used during Shuttle missions and on the Mir Space Station. More recently, several interval-type protocols have been routinely used on the International Space Station (ISS) since Expedition 1 (Loehr et al., 2015). The intensity of these treadmill-based protocols was initially limited by technological constraints (e.g., maximal belt speed); however, the availability of the “T2” treadmill and cycle ergometry protocols from Expeditions 20–25 onwards enabled exercise at higher intensities (Loehr et al., 2015). The maximum intensity of cycle-based protocols is currently 90% VO_2max_ – characterizing them as HIT rather than SIT. However, the within-session exercise intensity varies (60 to 90% VO_2max_), thereby differing from typical experimental HIT protocols where prescribed intensity within a session remains constant. Despite the routine use of interval exercise during spaceflight, NASA’s SPRINT study (National Aeronautics and Space Administration [NASA], 2018) is the only controlled investigation involving HIT in μG to date. Notwithstanding positive initial findings, the experimental design and limited available data from this study (Goetchius et al., 2019) make it impractical to draw definitive conclusions about the effectiveness of this training approach.

Maximal intensity exercise, in the form of maximal oxygen uptake (VO_2max_) assessment, was first incorporated during Shuttle Missions (Levine et al., 1996; Moore et al., 2001) with tests performed on ISS since 2009 (Moore et al., 2014) and used operationally without incident since 2016. This could provide a framework for the use of HIT at intensities up to 100% VO_2max_ during flight, which have been delivered with low risk across a range of healthy and clinical terrestrial populations (Rognmo et al., 2012). While SIT protocols (≥100% VO_2max_) may represent low risk in terrestrial populations, the physiology of astronauts is altered (although not apparently compromised, e.g., maximum heart rate; Moore et al., 2014) in microgravity, and therefore, the use of SIT for countermeasure exercise requires additional consideration.

Although HIT session duration is often ≥30 minutes, this is consistent with current continuous and interval-type protocols used on ISS and would fit within the current time allowance for aerobic exercise (60 min) (Loehr et al., 2015). However, as HIT achieves significant benefits when interval duration and/or number is reduced, time savings may well be realized. Moreover, if HIT can induce neuromuscular changes, this reduces current and future reliance on resistance training, potentially achieving further time savings. In addition to potential time savings, lower energy expenditure and elevations in metabolism from HIT compared with continuous protocols (Matsuo et al., 2012) offer significant operational benefits over the course of a long mission. Specifically, reduced energy requirements (i.e., provision of food, which represents additional mass) and reduced burden on the environmental management systems (i.e., removal of CO_2_, moisture, heat). The effectiveness of short-term low-volume HIT programmes might also facilitate the intermittent use of countermeasure exercise to achieve further savings in resources and by-product management. In this approach, informed by systematic tests of function (e.g., VO_2max_), a degree of adaptation could be allowed with periods of HIT interspersed to manage the magnitude of change.

Finally, the potential effectiveness of HIT across different exercise modes offers an advantage for exploration. Vehicle/mission constraints make it likely that only one exercise device will be available to crew and current concepts do not include treadmill running (National Aeronautics and Space Administration [NASA], 2017; The Danish Aerospace Company, 2018). However, they do envisage multiple modes of exercise, including cycling, rowing, and upper- and lower-body resistance-type exercise, all of which could accommodate HIT/SIT protocols.

## Conclusion

Collective evidence suggests that HIT could offer a range of operational and physiological benefits during spaceflight making it a viable tool within the exercise countermeasure programme. Substantial terrestrial findings support the efficacy of HIT as a time-efficient tool for cardiorespiratory fitness improvement with emerging data indicating potential beneficial effects on the neuromuscular system. The potential for HIT to impact other physiological markers affected by μG (e.g., BMD) remains largely unknown however and further investigation is warranted. Furthermore, despite encouraging terrestrial evidence, there remains no rigorous evaluation of HIT in μG and the efficacy of HIT during spaceflight is still unknown. Finally, consideration of astronaut-specific physiology (e.g., μG-induced fluid shifts) as well as logistical constraints (e.g., provision of appropriate exercise devices) and exercise programming variables is needed to maximize the potential application of HIT.

## Author Contributions

All authors listed have made a substantial, direct and intellectual contribution to the work, and approved it for publication.

### Conflict of Interest Statement

JS is employed by KBRwyle GmbH, Cologne, Germany.

The remaining authors declare that the research was conducted in the absence of any commercial or financial relationships that could be construed as a potential conflict of interest.
